# Cold Consolidation of Pharmaceutical Waste Glass Powders Through Alkali Activation and Binder Jet 3D Printing

**DOI:** 10.3390/ma17215164

**Published:** 2024-10-23

**Authors:** Hamada Elsayed, Filippo Gobbin, Alberto Barci, Enrico Bernardo, Paolo Colombo

**Affiliations:** 1 Department of Industrial Engineering, University of Padova, 35131 Padova, Italy; filippo.gobbin@unipd.it (F.G.); alberto.barci@studenti.unipd.it (A.B.); enrico.bernardo@unipd.it (E.B.); 2 Department of Materials Science and Engineering, Pennsylvania State University, University Park, PA 16801, USA

**Keywords:** borosilicate glass, alkali-activated materials, cold consolidation, waste glass upcycling, binder jetting

## Abstract

The recent COVID-19 emergency has led to an impressive increase in the production of pharmaceutical vials. This has led to a parallel increase in the amounts of waste glass; manufacturers typically recover material from faulty containers by crushing, giving origin to an unrecyclable fraction. Coarse fragments are effectively reused as feedstock for glass melting; on the contrary, fine powders (<100 microns), contaminated by metal and ceramic particles due to the same crushing operations, are landfilled. Landfilling is also suggested for pharmaceutical containers after medical use. This study aims at proposing new opportunities for the recycling of fine glass particles, according to recent findings concerning alkali activation of pharmaceutical glass, combined with novel processing, i.e., binder jetting printing. It has already been shown that pharmaceutical glass, immersed in low-molarity alkaline solution (not exceeding 2.5 M NaOH), undergoes surface dissolution and hydration; cold consolidation is later achieved, upon drying at 40–60 °C, by a condensation reaction occurring at hydrated layers of adjacent particles. Binder jetting printing does not realize a full liquid immersion of the glass powders, as the attacking solution is selectively sprayed on a powder bed. Here, we discuss the tuning of key parameters, such as the molarity of the attacking solution (from 2.5 to 10 M) and the granulometry of the waste glass, to obtain stable printed blocks. In particular, the stability depends on the formation of bridges between adjacent particles consisting of strong T-O bonds (Si-O-Si, Al-O-Si, B-O-Si), while degradation products (concentrating Na ions) remain as a secondary phase, solubilized by immersion in boiling water. Such stability is achieved by operating at 5 M NaOH.

## 1. Introduction

The COVID-19 pandemic has significantly amplified the demand for pharmaceutical containers, specifically vials to store and transport large numbers of vaccines, requiring the use of proper packing materials to maintain the airtightness and efficiency of the vaccines contained [1]. Glass, compared to metals and ceramics, offers superior qualities when it is used as a packaging material. It has great resistance to liquid corrosion and gas permeability, ensuring the safe storage and the vaccine integrity over time, as well as high transparency, which allows for the easy visual inspection of the vial contents, which is important for quality control and monitoring the condition of the content. It can also be versatilely formed into a variety of shapes to meet several package requirements and customized applications. Among different types of pharmaceutical glasses, borosilicate glass is particularly valuable, especially in terms of thermal and chemical stability [2,3]. These characteristics make it ideal for preserving sensitive compounds such as vaccines, which require consistent conditions to maintain their efficiency, without showing signs of surface deterioration and contamination.

The increased use of pharmaceutical glass, prompted by the mass production and distribution of billions of COVID-19 vaccines, has increased pharmaceutical glass waste. As a result, the amount of pharmaceutical glass that can be recycled has increased, raising awareness about the importance of recycling/upcycling pharmaceutical glass waste. In particular, glass can be recycled in two routes: a closed-loop technique, which involves melting the glass to create new products similar to the originals, and an open-loop approach, which produces items with distinct features and applications. When the items obtained have a higher economic worth than the original material, the process is referred to as upcycling [4]. End-of-life containers cannot be easily recycled into new products. However, glass can be recycled endlessly through melting. However, this process faces numerous challenges in practice: fragmented glass cannot be separated by color, and the decolorization process is extremely difficult to perform. Furthermore, metal and ceramic contaminations can lower the quality of recycled glass when compared to glass made from mineral feedstock, and removing these impurities is a difficult operation [5,6]. Therefore, efficient recycling methods are essential to managing the increasing volume of waste while also ensuring a sustainable approach to minimizing environmental impact.

One frequently explored approach is the production of alkali-activated materials (AAMs), which have the potential to replace Portland cement (OPC) [7,8]. AAMs can achieve high mechanical strength values, and are considered to be eco-friendly materials with a significantly lower CO_2_ footprint compared to OPC while delivering comparable performance [9]. Waste glass has been employed in geopolymer manufacturing both as an aggregate [10,11] and also as the primary material for activation due to its high silica content [12]. Its large surface area, resulting from the irregular shape of the grains, and its amorphous structure enable the rapid activation of glass waste surfaces in alkaline conditions [13]. The alkali activation process involves the dissolution of the aluminosilicate structure of the material in a basic solution, which releases hydrated oligomers composed of Si^4+^ and Al^4+^ ions interconnected together later by bridging oxygen [14]. In addition, Avram et al. showed that an alkaline solution facilitates the dispersion of small aluminosilicate particles, preventing their sedimentation, which is considered a requirement for geopolymer structure formation [15].

Borosilicate glasses constitute a class of oxide glasses primarily composed of two main network-forming oxides: silicon dioxide (silica, SiO_2_) and boron trioxide (boria, B_2_O_3_). These glasses also include network modifiers, such as alkali and alkaline earth oxides. Additionally, they often contain proportions of intermediate oxides, such as aluminum oxide (alumina, Al_2_O_3_) and other oxides [16].

The alkali activation of boro-alumino-silicate pharmaceutical glass is expected to produce a zeolite-like gel through the bridging of [SiO_4_] and [AlO_4_] tetrahedra, similar to geopolymers. Specifically, mild alkali activation using low-molarity alkaline solutions (such as NaOH and KOH) causes partial surface dissolution of the glass powder. Upon aging and curing, this results in the bonding of adjacent unreacted particles through thin surface layers. Adjacent particles are bound together by the room temperature condensation of Si–OH, B–OH, and Al–OH terminal groups at the surface, forming hydrated alkali alumino-silicate zeolite-like gels. Additionally, high boron oxide content is advantageous as it promotes the formation of zeolite-like networks with [BO_4_] units. These gels can also act as binders between the remaining glass powders, facilitating cold consolidation sintering without the need for any firing process [17].

The purpose of the current study was to investigate the feasibility of upcycling borosilicate pharmaceutical glass, starting with fine glass powder and an alkaline solution, to produce various porous ceramics through alkali activation and additive manufacturing.

Additive manufacturing (AM), commonly known as 3D printing, refers to technologies that create physical objects from 3D model data by successively adding material layer by layer, in contrast to subtractive manufacturing and formative manufacturing methodologies [18]. Additive manufacturing (AM) techniques are a series of methods increasingly studied because they could enable the fabrication of objects with complex geometries in a simpler way compared to conventional shaping methods. These technologies are currently used for various applications in various engineering industries. According to the American Society for Testing and Materials (ASTM) [19], AM technologies are categorized into different process types: binder jetting (BJT), material extrusion (MEX), directed energy deposition (DED), material jetting (MJT), powder bed fusion (PBF), sheet lamination (SHL), and vat photopolymerization (VPP).

Binder jetting and material extrusion printing are well-known technologies developed to enable large-scale additive manufacturing (AM) using different binders. In particular, in recent years, the production of construction material has been explored using powder bed-based additive manufacturing technology, specifically binder jetting (BJT), employing different sustainable inorganic binders such as calcium aluminate cement and Portland cement [20], alternative cements [21,22], and geopolymers [23].

This paper presents a potential alternative method for glass recycling that takes advantage of the binder jetting 3D printing technology. This study explores the use of borosilicate glass powders as reactive powder bed materials for powder bed 3D printing. The process involves selectively activating the powder bed by directly jetting sodium hydroxide solutions, eliminating the need for vigorous mechanical mixing, which is commonly required in the manufacturing of conventional alkali-activated materials. The binder jetting process utilizes a large-volume printer with a cubic voxel resolution of 3.0 × 3.0 × 3.0 mm^3^, producing large alkali-activated objects for various potential applications. This method produces geopolymer-like materials of significant dimensions, demonstrating that the process represents an economically viable route for valorizing discarded pharmaceutical glass.

## 2. Materials and Methods

The Pharmaceutical borosilicate glass (BSG) sourced from crushed waste vials supplied by Stevanato Group (Piombino Dese, Padova, Italy) was used as the starting material. The glass had already been ground by the company, resulting in the particle size distribution shown in Table 1. This distribution was measured by passing the glass powder through sieves with progressively smaller mesh sizes and measuring the remaining powder on each sieve. All of the samples analyzed in this study were produced without further processing of the glass powder. The glass powder was therefore used for building the powder bed via the binder jetting process. The chemical composition of the glass is as follows: 72 wt.% SiO_2_, 7 wt.% Al_2_O_3_, 12 wt.% B_2_O_3_, 6 wt.% Na_2_O, 2 wt.% K_2_O, 1 wt.% CaO, and <0.1 wt.% BaO. Sodium hydroxide, dissolved in tap water, was used as an alkaline solution acting as a binder.

To investigate the effect of sodium hydroxide molarity on the success of alkaline activation and the stability of the resulting alkali-activated materials, small samples were created manually in the first phase by jetting sodium hydroxide solutions with three different molarities (2.5 M, 5 M, and 10 M) onto the glass powder using a pipette to simulate the printing process. These solutions were prepared by mixing the appropriate amount of sodium hydroxide with water for 12 h. Then, the large samples were made using a large-volume 3D binder jetting printer employing two different formulations: in the first one, the powder bed was composed entirely of ground glass powder, while in the second formulation, the powder bed consisted of 98% ground glass powder and 2% additive (an organic superabsorbent sourced from BASF that is commercially available for use in cement applications) to help retain the activation solution and prevent excessive diffusion, as well as to control the printing accuracy and quality.

**Figure 1:**
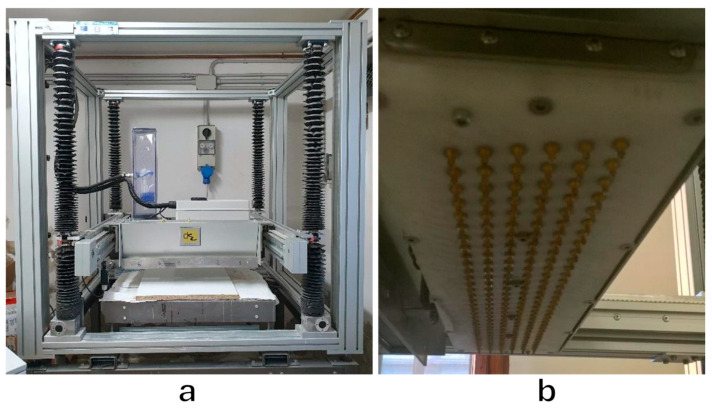
(**a**) Overall view of the employed binder jetting printer. (**b**) Close-up of the printhead, which features 192 nozzles with an internal diameter of 1.1 mm.

The 3D printer used in the current research was developed by Desamanera (Rovigo, Italy), and is capable of producing large-sized pieces, up to a maximum of 600 × 600 × 600 mm^3^, with a cubic voxel resolution of 3.0 × 3.0 × 3.0 mm^3^. A recoating system is used to generate the powder bed, which is deposited in the x–y plane. A specific volume of the required layer (50% volume, i.e., half a layer with a thickness of 1.5 mm) is deposited while the printer head moves forward. In the meantime, the printer head sprays liquid (the binder) onto selected areas of the powder bed layer, based on the printed part’s designed geometry, through all or some of the 192 nozzles in it, which have an internal diameter of 1.1 mm (see Figure 1). Subsequently, the printer head moves backward (after being raised in the z direction), and the recoating system finally deposits the remaining powder to create a total layer thickness of 3 mm. This process is repeated layer by layer until the printing process is complete. The printer components were specifically designed by Desamanera to work with inorganic binders such as water and alkaline solutions with high pHs and viscosities greater than water.

To achieve the binder jetting of the BS glass, the nozzle opening time was fixed at 25 ms, and the printing speed was maintained at 30 mm/s in both directions. The same parameters were used to print both glass powder bed formulations: one with a powder bed with 100 wt% borosilicate glass, and the other with a bed of 98 wt% glass and 2 wt% additive. The additive was incorporated into the powder bed to assess whether it could help the printed pieces maintain the desired dimensions.

The printing was performed at room temperature with a humidity range of 50–60%. After printing, the samples were left to cure inside the printing bed overnight, and later they were further aged and cured at 60 °C for 7 days. Then, the samples were extracted, cleaned, and prepared for the subsequent tests.

Morphological and microstructural characterizations of the printed samples were carried out at various magnifications using optical stereomicroscopy (AxioCam ERc 5 s Microscope Camera, Carl Zeiss Microscopy, Thornwood, NY, USA) and scanning electron microscopy (FEI Quanta 200 ESEM, Eindhoven, The Netherlands).

Fourier-transform infrared spectroscopy (FTIR) was performed to analyze the bond structures of the borosilicate glass before and after alkaline activation through the binder jetting process. Spectra were recorded in the 4000–400 cm^−1^ range using an ATR-FTIR spectrometer (Jasco FT/IR-4200 type A, Easton, MD, USA).

The identification of the crystal phase was achieved by means of X-ray diffraction (XRD) on powdered samples (Bruker AXS D8 Advance, Bruker, Germany). X-ray diffraction patterns were recorded over a 10–70° 2θ range using CuKα radiation at 40 kV and 40 mA, with a step size of 0.05° and step time of 2 s. The phase identification was recognized using the Match! program package (Crystal Impact GbR, Bonn, Germany), with data from the PDF-2 database (ICDD-International Centre for Diffraction Data, Newtown Square, PA, USA).

The bulk density was calculated using the ratio of the mass to the bulk volume. The mass of the samples was measured using a laboratory balance, and the bulk volume was determined from the dimensions of the sides of the samples (cube-shaped or parallelepiped-shaped) measured with a digital caliber. The apparent density was determined using a helium (He) pycnometer (Micromeritics AccuPyc 1330, Norcross, GA, USA) by inserting solid printed parts into the instrument, while the true density was obtained by inserting the same pieces into the instrument; however, afterward, they were grounded and sieved to below 36 µm. Then, the total porosity of the printed structures was therefore calculated as one minus the ratio between their bulk to their true powder density, whereas the open porosity was calculated as one minus the ratio between the bulk density and the apparent density. Since the open and total porosity values were found to be very similar in all cases, this paper will refer only to the total porosity.

The mechanical properties were assessed using a universal testing machine (Quasar 25, Galdabini S.p.a., Cardano al Campo, Italy). Bars with dimensions of 13 × 2.5 × 2.5 cm^3^ were cut from the printed pieces and subjected to three-point bending strength tests. Subsequently, cubes with side lengths of 2.5 cm were obtained from these bars, on which compression strength measurements were performed. All of the tests were conducted at a loading speed of 1 mm/min. A total of 10 measurements were conducted per each specimen and testing condition.

## 3. Results

**Figure 2:**
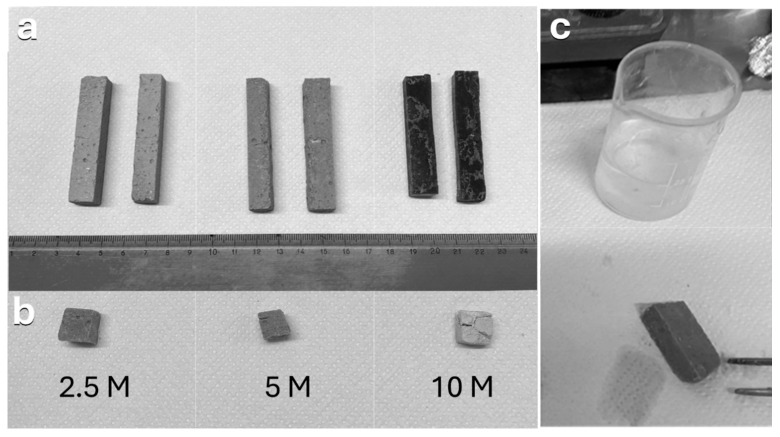
(**a**) Hand binder-jetted borosilicate parts with different concentrations of NaOH solution; (**b**) the printed parts after a 2 h boiling test and complete drying; (**c**) the wet-printed part after the boiling test, along with the water used for the test.

To assess the reactivity of borosilicate pharmaceutical glass to alkali activation for large-scale binder jetting processes, small samples were initially prepared by activating glass powder with sodium hydroxide solutions using three different molarities (2.5 M, 5 M, and 10 M). Figure 2a shows the hand binder-jetted parts after the alkali activation and consolidation processes. Specifically, the interaction between the alkaline solution and glass powder leads to a partial dissolution of the glass, forming a geopolymer-like gel that increases the system’s viscosity over time. This helps to prevent enhanced binder spreading (unwanted binder bleeding) inside the powder bed. The formation of a gel phase led to cold consolidation after hardening over time. This demonstrated the excellent reactivity of the borosilicate glass powder in forming a geopolymer-like gel, which facilitated the fabrication of 3D objects with sufficient resolution and adequate mechanical strength.

To observe the differences between samples prepared using sodium hydroxide solutions of different molarities, the produced samples were subjected to a boiling test and were observed under SEM.

Initially, a feasibility study was required to evaluate the minimum concentration of sodium hydroxide as an alkaline activator and to verify whether the consolidated material could withstand a boiling water test, demonstrating its durability and stability after the binder jetting process. Consequently, the binder-jetted parts, activated using different sodium hydroxide molarities, were immersed in boiling water for 2 h. The boiling tests showed that none of the samples dissolved in boiling water after 2 h, confirming their resistance to hydrolytic degradation and maintaining their structural integrity without deformation, indicating the formation of a stable geopolymer-like material at the interface between the glass particles. This was evidenced by the clarity of the remaining water and the condition of the wet-printed parts after the boiling test, as shown in Figure 2c. However, as observed in Figure 2b, only the samples activated with the 2.5 M and 5 M sodium hydroxide solutions fully passed the boiling test, showing no superficial cracks after drying, while the sample activated with a 10 M sodium hydroxide solution exhibited significant swelling and prominent cracks after drying. This behavior is likely due to the presence of residual sodium hydroxide, which reacts with water, causing swelling.

**Figure 3:**
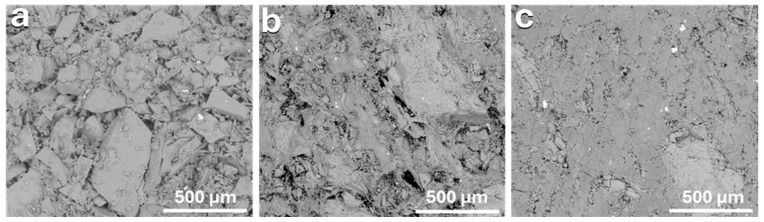
SEM images of the microstructure of hand binder-jetted borosilicate glass powder using different sodium hydroxide concentrations: (**a**) 2.5 M NaOH; (**b**) 5 M NaOH; (**c**) 10 M NaOH.

Notable differences in the microstructures of the cold consolidated binder-jetted samples prepared with NaOH solutions of varying molarities could be observed (see Figure 3). The matrix became more compact as the molarity of the sodium hydride solution increased, due to greater dissolution and interaction at the glass particle surfaces. As a compromise between the boiling test and SEM investigation, it was decided to use a 5 M NaOH solution for large-volume binder jetting.

**Figure 4:**
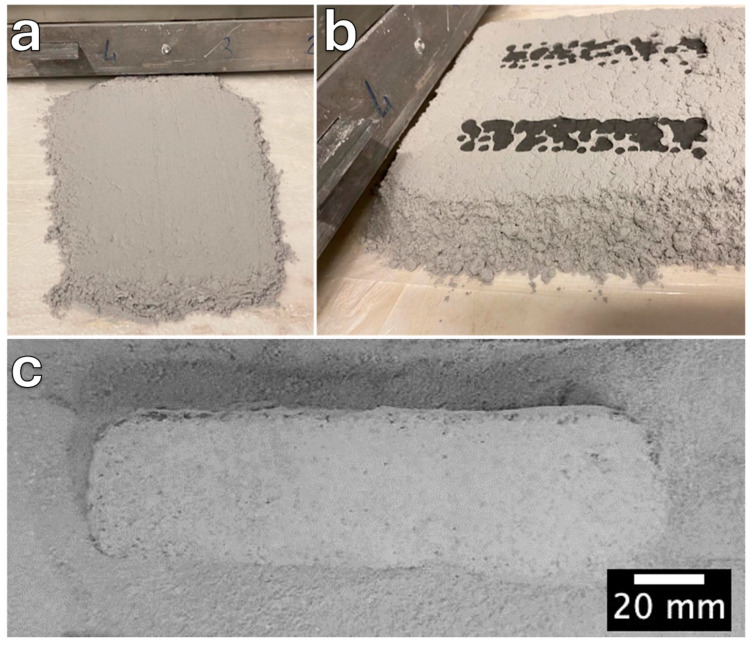
Large-volume binder jetting of borosilicate glass: (**a**) creation of a smooth and homogeneous powder layer; (**b**) selective jetting of NaOH binder; (**c**) consolidated printed parts during the depowering process and extraction from the powder bed.

Figure 4 illustrates the large-volume binder jetting process that was carried out, which generally consists of several steps: (1) deposition of the borosilicate glass powder through a recoating system to create a homogeneous smooth powder bed layer in the x–y plane, as shown in Figure 4a; (2) selective jetting of the 5 M sodium hydroxide as the activation binder (Figure 4b), followed by immediate recoating of the printed layer; (3) repeating the powder deposition and binder jetting layer-by-layer until the part is complete; and (4) extracting (depowering) the printed part after complete cold consolidation, as shown in Figure 4c.

**Figure 5:**
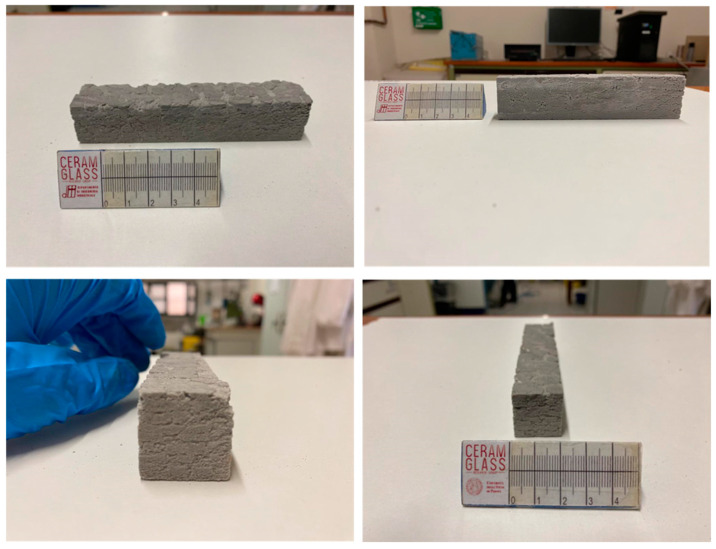
The printed bar, viewed from different directions to confirm the integrity of the printed parts and the cold consolidation of borosilicate glass through alkali activation and binder jetting.

The overall morphology of the printed bar (135 × 30 × 30 mm^3^) is shown in Figure 5. The figure presents the top and side views of the printed bar from different directions after cleaning unreacted glass particles following extraction from the powder bed, as well as images of the cut surfaces. These surfaces reveal a strong interface between the printed layers, confirming the formation of a geopolymer-like gel during consolidation.

**Figure 6:**
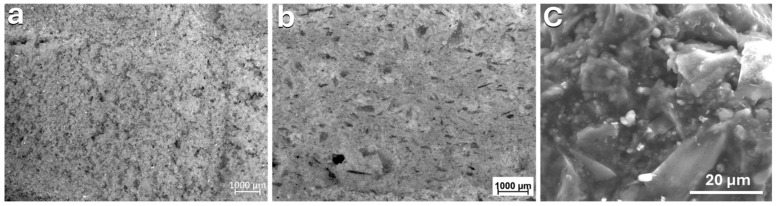
(**a**) Morphology of the powder bed layer; (**b**) morphology of the binder-jetted part; (**c**) microstructure of the printed part.

To understand the formation of the geopolymer-like gel matrix that enhances adhesion between glass particles and enables cold consolidation of the printed parts during binder jetting, we investigated the morphology of the powder bed after layer deposition and the surface of the consolidated binder-jetted parts. Figure 6a shows that the powder bed was deposited as an almost smooth layer with good powder distribution, exhibiting limited defects and residual porosity between the glass particles. The optical image in Figure 6b shows the microstructure of the printed samples and demonstrates the formation of a gel that rapidly hardened, facilitating the effective interaction between the borosilicate glass particles and enabling high-resolution cold consolidation with reduced porosity compared to the powder bed. The SEM image in Figure 6c further confirms the dissolution of the borosilicate glass powder after alkali activation during binder jetting and the formation of a geopolymer-like gel phase. This gel phase leads to the development of a binding matrix that consolidates the printed parts by binding the unreacted particles together.

To better monitor the formation of the geopolymer-like gel during binder jetting and the cold consolidation of BSG, we investigated the bond structure within the samples using FTIR. Spectra were recorded for ground non-activated as-received BSG powder, as well as for 3D-printed parts after binder jetting, both in their green (as printed) state and after undergoing the boiling test.

**Figure 7:**
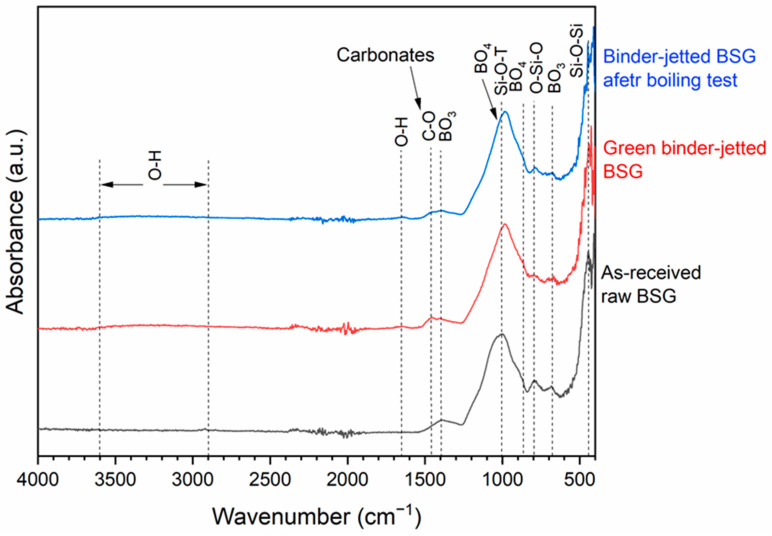
FT-IR spectrum of the as-received, green binder-jetted, and post-boiling borosilicate glass.

Figure 7 presents the FT-IR spectrum of the as-received borosilicate glass, highlighting its key characteristic peaks. Notably, the peak around 1400 cm^−1^ is attributed to the symmetric stretching relaxation of the B-O bond in trigonal BO_3_ units. The broad absorption band observed around 1005 cm^−1^ is linked to the asymmetric stretching vibrations of Si-O-T (T = Si or Al) bonds, as well as to both BO_3_ and BO_4_ groups. The peak at approximately 795 cm^−1^ is associated with the symmetric stretching vibration of the O-Si-O bond. The band at about 670 cm^−1^ corresponds to the bending vibration of bridging oxygen (B-O) between trigonal BO_3_ groups. Lastly, the broad band centered at 460 cm^−1^ corresponds to the Si-O-Si bending vibration [24].

Comparing the FTIR spectra of the as-received borosilicate glass with the binder-jetted material, both in its green state and after boiling, we can observe that although the spectra are similar, significant changes can be pointed out, indicating that a structural change occurred in the material due to alkali activation during jetting. Specifically, in the 3D-printed samples, the peak around 1005 cm^−1^, which corresponds to the tetrahedral stretching modes of the Si-O-T (T = Si or Al) bond in the raw BSG powder before activation, shifts to approximately 980 cm^−1^. This shift suggests a structural change due to the replacement of SiO_4_ units with AlO_4_ during gelation, which resulted from a decrease in the Si/Al ratio at the tetrahedral Si sites during the formation of a geopolymer-like gel. After alkali activation, more Al atoms formed in a tetrahedral coordination bond with Si atoms via oxygen bridges, promoting the formation of binder gels such as sodium aluminosilicate hydrate (N-A-S-H). This process leads to a shift of the Si-O-T bond’s vibration band to lower wavenumbers [23,25]. This effect is also evident in the shift and decreased intensity of the Si–O–Si bending peak around 800 cm^−1^ following alkali activation, suggesting the formation of a hydrated gel. Additionally, the peak around 1005 cm^−1^ exhibited asymmetry, likely due to the B-O bond in BO_3_ trigonal units, while the improved symmetry of this peak in the printed sample could be attributed to an increased amount of BO_4_ tetrahedral units. The B-O stretching vibrations in BO_3_ trigonal units correspond to a band around 1200 cm^−1^, whereas in BO_4_ tetrahedral units, they correspond to a band at about 900 cm^−1^ [26,27]. Additionally, alkali activation led to an enhancement of the band at 880 cm^−1^, which is associated with the stretching vibrations of B–O bonds in BO_4_ tetrahedra. Consequently, alkali activation generally promotes the development of highly durable gels with a “zeolite-like” network structure formed by the bridging of SiO_4_, AlO_4_, and BO_4_ units. In fact, the glass particles in the printed samples became inherently well-integrated through alkali activation during the binder jetting process.

Moreover, unlike the raw BSG spectrum, the 3D green printed samples exhibited broad bands within the 3100–3600 cm^−1^ range and a band centered at 1645 cm^−1^, which can be attributed to the stretching and bending vibrations of O-H bonds related to water molecules. These molecules are either surface-absorbed or trapped in the large cavities of the geopolymer framework. This suggests the presence of a hydrated structure, similar to what is typically observed in alkali-activated materials.

Regarding the other peaks, the peak at 1460 cm^−1^, detected after binder jetting with NaOH, can be attributed to the stretching vibrations of the C-O bond, indicating atmospheric carbonation. Excess sodium ions in the alkali-activated matrix may have reacted with CO_2_ to form sodium bicarbonate or hydrated carbonate [28]. This was not observed after boiling the printed samples, likely due to the removal of soluble carbonates by the washing effect, which left only the strong bonds between the particles, indicating that the glass particles possessed robust connections. Overall, except for these soluble components, the FT-IR spectra of the printed BSG before and after the boiling test were practically identical.

**Figure 8:**
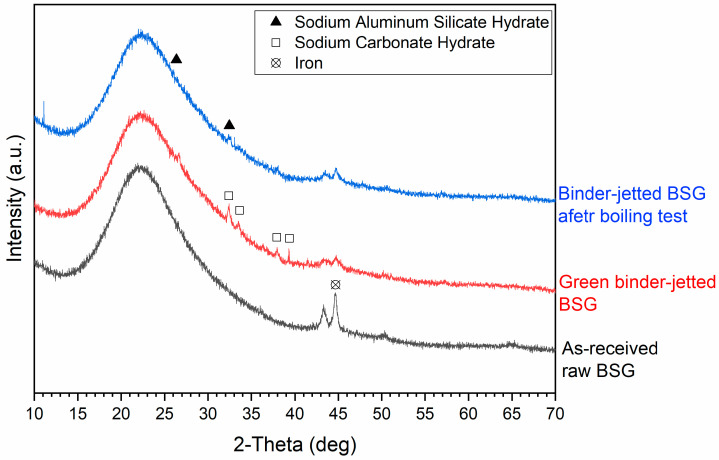
X-ray diffraction patterns of the as-received, green binder-jetted, and post-boiling borosilicate glass.

Figure 8 reports the X-ray diffraction patterns of borosilicate glass before and after binder jetting. The XRD pattern of the as-received pharmaceutical glass powder indicates its amorphous structure, as expected. Binder jetting, after activation by 5 M NaOH, led to the formation of a geopolymer-like gel. While geopolymers are generally characterized by an amorphous structure, the co-polymerization of individual alumino and silicate species can lead to the formation of amorphous to semi-crystalline geopolymers [29,30,31]. In the spectra, a slight shift of the hump from approximately 21–22° towards higher 2θ values is observed, indicating the formation of a geopolymer-like gel. The formation of a sodium alumino-silicate hydrate ([PDF#03-0234], Gmelinite zeolite phase) has also been confirmed, along with a hydrated sodium carbonate phase ([PDF#76-0910], Na_2_CO_3_·H_2_O), which disappeared after boiling [32,33]. This confirms that the binder jetting process of borosilicate glass, through alkali activation, resulted in the formation of durable gel with a “zeolite-like” network structure, created by bridging SiO_4_, AlO_4_, and BO_4_ units. From the SEM image, Figure 6c, it is also possible to observe the formation of zeolite crystals, with the typical structure reported in other studies [34,35].

A phase corresponding to iron [PDF#087-0721] was also observed in all three spectra with the peak intensity reduction upon the alkali activation process; this presence is likely a result of the glass milling processes within the company.

It was observed that the bar printed with the additive exhibits more linear dimensional accuracy, but only in the direction perpendicular to the binder jetting direction, compared to the bar printed without the additive. This reduction in accuracy could be attributed to the bleeding mechanism; at higher binder saturation levels, excess binder tends to spread beyond the edges of the printed sample, leading to decreased linear dimensional accuracy [36]. This suggests the presence of more horizontal binder spreading rather than vertical penetration, which aligns with the longer penetration time observed. These parameters depend not only on particle size distribution, but also on the physical properties of the binder and the wettability of the powder bed [37]. However, while the printed part with the additive shows better linear accuracy, its stability, as measured through a boiling test, was significantly worse. In the case of the bar without the additive, the integrity of the printed sample and the clarity of the remaining water after the boiling test indicate that geopolymerization occurred successfully. In contrast, the bar printed with the additive completely disintegrated in the water, likely because the excessive retention of the solution prevented the formation of sufficient (geo)polymeric bonds.

To evaluate the mechanical performance of binder-jetted alkali-activated BSG components, mechanical tests, in terms of compressive and bending strength, were conducted one week after printing. The compressive strength was tested both along the binder jetting direction (X-direction) and perpendicularly to it (i.e., the layer-assembling direction, Z-direction). The results in Table 2 show that all printed samples exhibited porosity values slightly below 60 vol%. Binder jetting products generally exhibit higher porosity compared to those produced by other additive manufacturing techniques due to the powder bed-based process. This porosity can be influenced by the characteristics of the powder bed particles, powder–binder interactions and reactions, as well as post-processing infiltration [38].

For the glass samples printed with the additive, the measured strength values were slightly higher than those of the samples without the additive, despite being less chemically stable, as observed in the previously described boiling test. The use of the additive resulted in chemically unstable samples. Therefore, we did not further investigate the final properties of the samples obtained using it.

The compressive strength of the printed sample, containing only glass in the powder bed without any additives, ranged from 0.77 to 1.00 MPa, depending on the loading direction. Although the printed samples exhibited anisotropic mechanical behavior, the similar strength values across different loading directions suggest a relatively homogeneous microstructure with a good interface between the printed layers.

Combining the measured compressive strength with the relative density and porosity values of the binder jetting glass components, the results from the current work indicate that cold consolidation of BGS is achievable without any post-process firing. Notably, these results are also comparable to those from previous studies on the same alkali-activated glass, without requiring the vigorous mechanical mixing typically used in conventional alkaline activation processes. For instance, porous structures printed using direct ink writing of alkali-activated pharmaceutical glass waste (BSG), with the addition of 20 wt% TiO_2_ nanoparticles, had a total porosity of 65% and a compressive strength of 0.3 MPa [17].

Similarly, previous research focused on printing geopolymer-based parts using a powder bed composed of sodium metasilicate, sand, and varying proportions of slag and fly ash as geopolymer precursors, ranging from 100% slag to 0%, with an aqueous commercial solvent as a binder. The printed geopolymer samples exhibited a porosity of around 58% and compressive strength, ranging from 0.24 to 0.91 MPa, depending on the testing direction and fly ash content. However, the mechanical strength after binder jetting was adequate for the de-powdering process during extraction from the powder bed. A post-processing strategy was employed for further strength enhancement [39,40].

In summary, boro-alumino-silicate pharmaceutical glass powders present novel opportunities for creating stable and consolidated porous alkali-activated components using binder jetting additive manufacturing. This method facilities the advantages of additive manufacturing to fabricate complex structures with well-defined details. Additionally, the environmentally friendly gels developed through alkali activation have demonstrated functional properties, such as successful dye adsorption, highlighting their potential in emerging environmental technologies [17].

## 4. Conclusions

In this work, a large-scale binder jetting technique was successfully utilized to manufacture a 3D-printed alkali-activated structure. Selective jetting of an alkaline liquid (5 M aqueous sodium hydroxide solution) onto BSG powder resulted in a hardened geopolymer-like material. The 3D-printed parts were developed through the cold consolidation of borosilicate glass powder sourced from recycled commercial pharmaceutical glass vials. The printed parts were found to be highly porous, with a porosity of approximately 60%, and the compression resistance values were comparable to those of porous parts and foams produced in previous studies using the same glass. These results show that the process represents a potentially economically viable way of valorization of discarded pharmaceutical glass for different applications.

Future work should focus on the following two aspects: reducing the diffusion of the binder solution into the powder bed to produce more precise-shaped parts without compromising the stability of the polymer network and reducing porosity to achieve higher mechanical properties. Both aspects are crucial to the binder jetting process, as they determine the development of printed materials and their characteristics for potential applications in various industries.

## Figures and Tables

**Table 1:** Particle size distribution (PSD) of borosilicate glass (BSG) powder.

Mesh Size (µm)	Weight (%)
>500	10.2
>420	3.40
>300	3.35
>200	8.10
>100	21.95
>63	16.35
>36	12.85
<36	21.90

**Table 2:** Physical and mechanical properties of 3D-printed glass components at 7 days after the binder jetting process.

Sample Name	Bulk Density (g/cm^3^)	Total Porosity (vol%)	Compressive Strength (MPa)		Bending Strength (MPa)
			Z-Direction	X-Direction	
Only glass	0.95 ± 0.02	59.45 ± 1.03	0.77 ± 0.26	1.00 ± 0.04	0.23 ± 0.01
With additive	0.98 ± 0.03	57.39 ± 1.32	1.15 ± 0.23	1.11 ± 0.14	0.35 ± 0.01

## Data Availability

The data presented in this study are available upon request from the corresponding author.
